# RetS-mediated environmental sensing coordinates TetR-dependent regulation of type III secretion system and virulence in *Pseudomonas syringae* pv. *actinidiae*

**DOI:** 10.1128/aem.00494-25

**Published:** 2025-06-10

**Authors:** Renjian Hu, Fu Zhao, Muhammad Asif, Jiaoyan Gu, Shuang Liang, Rong Fan, Youhua Long, Yong Wang, Fenghua Tian, Zhibo Zhao

**Affiliations:** 1 Department of Plant Pathology, College of Agriculture, Guizhou University 645736, Guiyang, China; 2 Research Center for Engineering Technology of Kiwifruit, Institute of Crop Protection, College of Agriculture, Guizhou University 645736, Guiyang, China; 3 Teaching Experimental Field of Guizhou University, Guizhou University 71206, Guiyang, China; Washington University in St. Louis, St. Louis, Missouri, USA

**Keywords:** bacterial canker, *Pseudomonas*, TetR/AcrR family, transcription factors, virulence, genetic regulation

## Abstract

Kiwifruit bacterial canker, caused by *Pseudomonas syringae* pv. *actinidiae* (Psa), is an emerging global concern in kiwifruit production. Successful Psa infection in kiwifruit relies on the type III secretion system (T3SS), which is governed by the HrpR/S-HrpL regulatory pathway. However, the mechanisms by which certain transcriptional factors regulate this pathway remain poorly understood. In this study, we identified the TetR/AcrR family transcription factors *C_22735* (*tetR1*) and *C_04700* (hybrid histidine kinase; *retS*) in the *P. syringae* pv. *actinidiae* (biovar 3) strain S26 using transposon libraries and a DNA pull-down strategy. Both the mutant strains *tetR1* and *retS* exhibited significantly reduced pathogenicity in kiwifruit leaf discs and a diminished ability to induce *hrpRS*, *hrpL*, and *hrpA* expression *in vitro* and showed a reduced hypersensitive response in *Nicotiana benthamiana*. Moreover, transcriptomic studies showed considerable overlap in downregulated genes between the *tetR1* and two-component system (TCS) *retS* mutant, highlighting the intertwined regulatory roles of both genes. This overlap suggests a tightly coordinated regulatory system, where *tetR1* serves as the response regulator of the hybrid histidine kinase *retS* to regulate T3SS and virulence. The electrophoretic mobility shift assays and luciferase-based promoter activity detection further revealed that TetR1 positively regulates the T3SS by directly binding to the promoter regions of *hrpR*/*S* and *hrpL*. This study identifies the *tetR1* gene as the first TetR-family transcription factor directly linking the hybrid TCS *retS* to HrpR/S-HrpL-mediated T3SS regulatory pathway activation in Psa for regulation.

**IMPORTANCE:**

This study uncovers the critical roles of two regulatory genes, *C_22735* (*tetR1*) and *C_04700* (*retS*), in *Pseudomonas syringae* pv. *actinidiae* (Psa) virulence via the type III secretion system (T3SS). Through genetic knockouts, complementation, and transcriptomic analyses, we establish *C_22735*, a TetR/AcrR family transcription factor, as a pivotal activator of T3SS-related genes essential for Psa’s virulence in kiwifruit. We further demonstrate that *retS*, a hybrid histidine kinase two-component system regulator, acts upstream by enhancing *tetR1* expression, forming a hierarchical network that drives the T3SS cascade during infection. *tetR1* serves as the response regulator of the hybrid histidine kinase RetS to regulate T3SS and virulence in Psa. These insights reveal how Psa orchestrates virulence to colonize kiwifruit tissues, advancing our understanding of bacterial plant interactions. Moreover, this regulatory framework offers promising targets for developing precise strategies to combat bacterial canker, mitigating its devastating economic toll on global kiwifruit production.

## INTRODUCTION

Bacterial diseases caused by *Pseudomonas syringae* pose a significant threat to a wide range of host plants, including economically important crops (1–3). Among its more than 60 pathogenic variants, *P. syringae* pv. *actinidiae* (Psa) causes bacterial canker of kiwifruit, a devastating disease that severely impacts global kiwifruit production (4, 5). Genomic and phylogenomic analyses have classified Psa into biovars 1, 2, 3, 5, and 6, with biovar 3 driving a worldwide pandemic (3, 6, 7). Central to Psa’s virulence is the type III secretion system (T3SS), a needle-like apparatus that delivers effector proteins into host cells to suppress immune defenses and promote pathogen proliferation (8–13). T3SS is encoded by a group of genes known as hypersensitive response (HR) and pathogenicity/hypersensitive response conserved (*hrp*/*hrc*) genes, which together form the T3SS cluster (8–13).

In Psa, as in other gram-negative bacteria, the T3SS is regulated by a complex network of transcriptional and post-transcriptional factors (14–20). Among these, the HrpR/S-HrpL pathway plays a pivotal role. HrpR and HrpS, enhancer-binding proteins that are encoded by a single *hrpRS* operon, form a heterodimer that activates transcription of *hrpL*, an alternative sigma factor that governs the expression of T3SS structural components and effectors (21), through the modulation of a conserved promoter motif known as the *hr*p-box (GGAACC-N15/16-CCACNNA) (22, 23). *hrpL* works with the alternative σ factor RpoN (σ^54^), as well as with HrpR and HrpS, to regulate T3SS gene expression (24). The circuitry of *hrp* regulation in *P. syringae* appears to involve a transcriptional activation cascade in which HrpR activates *hrpS*, HrpS activates *hrpL*, and HrpL activates transcription of the remaining *hrp* genes. Additionally, the bacterial AAA+ enhancer-binding proteins, HrpR and HrpS (HrpRS), activate σ^54^-dependent transcription at the *hrpL* promoter, triggering T3SS-mediated pathogenicity. The HrpR/S-HrpL regulatory cascade is modulated by various regulatory systems, including two-component systems (TCSs; RhpRS, CvsRS, and GacSA) and sigma factors, which collectively fine-tune T3SS activity in response to environmental and host cues (16–18). However, the specific contributions of individual regulators within this network, particularly in Psa, remain underexplored. As a structural component of the T3SS, HrpA is critical for both pilus assembly and regulatory functions. Mutations in *hrpA* disrupt T3SS assembly in *P. syringae* pv. *tomato* DC3000—similar to *hrpS* mutants—by significantly reducing transcript levels of the positive regulators *hrpR* and *hrpS*. This indicates that HrpA, as part of a supramolecular secretion structure, coordinates effector delivery, gene expression, and host-cell sensing during infection (16).

The TetR/ArcR type family of transcriptional regulators, widely distributed in bacteria and archaea, is a prominent group of one-component signal transduction systems (25). These proteins feature an N-terminal helix-turn-helix DNA-binding domain and a diverse C-terminal ligand-binding domain, enabling specific ligand interactions as dimers (26, 27). Named after the repressor TetR, they typically inhibit transcription by blocking RNA polymerase (25). However, some TetR members can act as activators or dual regulators (28, 29). TetR family regulators, beyond their typical repressor role, also control homeostasis, catabolic enzymes, and bacterial pathogenicity, including virulence (30, 31). A previous study by our group identified an OmpR-like transcription factor that negatively regulates *hrpR/S* transcription by binding to its promoter (32), while another recent work has uncovered two plant-derived TFs, AcREM14 and AcC3H1, which enhance disease resistance against bacterial canker in kiwifruit hybrid lines (33). The regulatory interplay between pathogen and host factors is exemplified by TCSs such as the hybrid sensory histidine kinase RetS and response regulator LadS. In *P. syringae* pv. *syringae*, RetS and LadS coordinate T3SS activity and other virulence factors. Similarly, in *Pseudomonas aeruginosa*, the RetS-LadS regulon governs diverse virulence mechanisms, including motility, biofilm formation (via exopolysaccharides), T3SS, and the type VI secretion system, all critical for pathogenicity across bacterial species (34, 35).

In this study, we employed genetic, biochemical, and transcriptomic analyses to elucidate the role of C_22735 (hereafter referred to as TetR1) in Psa virulence. Knockout mutants of *tetR1* and *retS* revealed their significant influence on T3SS expression, with *in vitro* assays demonstrating that *tetR1* enhances the promoter activity of *hrpR/S* and *hrpL*. These findings position TetR1 as a positive regulator of the HrpR/S-HrpL-T3SS cascade, dependent in part on *retS*. This study provides the first detailed insights into how TetR1 shapes Psa’s pathogenicity, offering a foundation for understanding the intricate transcriptional regulation of T3SS in this globally significant pathogen.

## RESULTS

### Screening and identification of novel transcription factor C_22735 reducing Psa infection

A transposon library was generated in the S26 strain to introduce random Tn insertions across the genome. One of the mutants exhibiting impaired pathogenicity was selected, and genome resequencing was performed to identify the precise location of the Tn insertion. This analysis revealed the insertion site at position 4,688,835, which disrupted the gene CN228-22735 (TetR1), encoding a TetR/AcrR family transcription factor, and a 569 bp gene encoded by a total of 189 amino acids. Separately, DNA pull-down assays were performed to investigate protein-DNA interactions, confirming that *tetR1* shows interaction with the promoters of *hrpR* and *hrpL* (data not shown). Further amino acid sequence BLAST analysis indicated that it is consistent across various Psa biotype populations and highly conserved within *P. syringae* eight genomospecies, differing by only one amino acid and showing over 99.9% similarity. In general, C_22735 is present in all members of the *P. syringae* complex species, revealing evolutionary trends with a similarity greater than 60% (Fig. S1). This strong evolutionary conservation of *tetR1* across *P. syringae* pathovars (e.g., sequence identity >90%) suggests that it is crucial for pathogen survival, likely through regulating core virulence or stress-response pathways (Fig. S2A). Comparative analysis of *tetR1* with other *P. syringae* members indicates that it is highly conserved at the N-terminus, which contains a helix-turn-helix structure. At the same time, the ligand recognition domain at the C-terminus shows significant variation (Fig. S2A). Additionally, the Ka/Ks value between *tetR1* in Psa and its homologous proteins ranges from 0.1 to 0.2 (Fig. S2B), suggesting that *tetR1* exhibits high functional stability throughout evolution. These findings imply that *tetR1* in Psa may possess a crucial conserved function due to homologs in other *P. syringae* strains. However, to our knowledge, no previous studies have specifically linked a TetR/AcrR family transcription factor in *P. syringae* or Psa to the direct regulation of T3SS genes, such as *hrpR/S* and *hrpL*. To investigate the role of TetR1 in host-pathogen interactions, we constructed Δ22735 (*tetR1* knockout), C22735 (complementation), and OE22735 (overexpression) strains in S26 and assessed their impact on pathogenicity in kiwifruit and HR induction in *Nicotiana benthamiana*

### TetR1 positively regulates T3SS expression in kiwifruit

**Fig 1:**
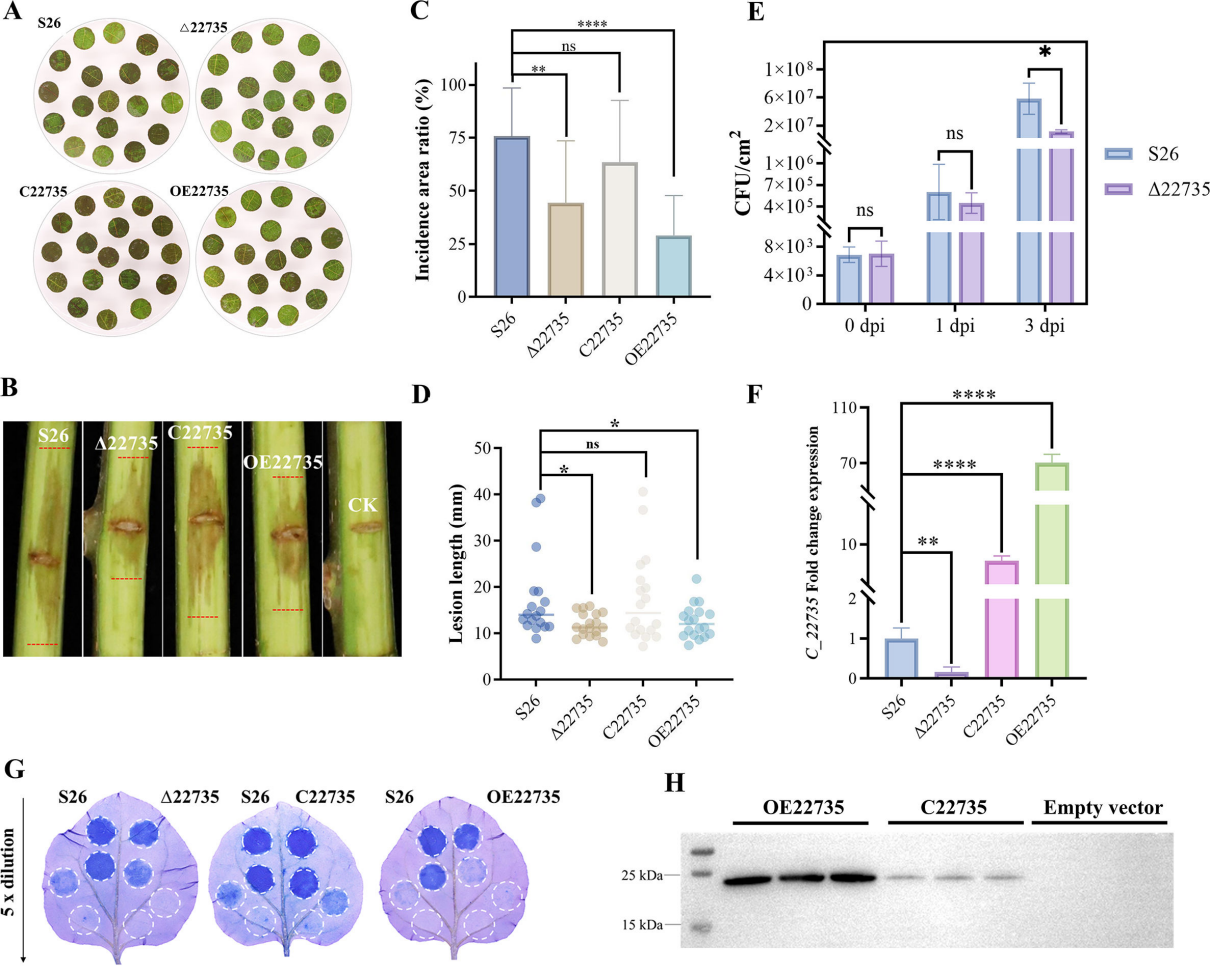
The role of *C_22735* (*tetR1*) in T3SS-mediated pathogenicity, HR, and protein expression. (**A**) The tested strains were infiltrated via immersion method on kiwi-fruit leaf discs, and necrosis symptoms on leaf discs were recorded after 5 d post-inoculation (dpi), and pathogenicity was recorded as disease symptoms (necrosis area) on kiwifruit leaves and branches with S26 WT control, Δ22735 (*tetR1* knockout), C22735 (complementation), and OE22735 (overexpression) strains in S26 (*n* = 18 per group). (**B**) Kiwifruit branches were wound inoculated (10 µL) to evaluate the pathogenicity of the constructed Δ22735 (*tetR1* knockout), C22735 (complementation), and OE22735 (overexpression) strains in S26, and the symptoms were recorded as area of necrotic spots on branch inoculation site (*n* = 18 branches per strain) after 10 dpi. (**C and D**) The calculated disease incidence area ratio on leaves and lesions length (mm) on kiwifruit branches were statistically compared using student’s *t*-tests; (*P < 0.05, **P < 0.01, and **P < 0.001). (**E**) S26 (WT) and Δ22735 strains were subject to bacterial counts using CFUs for 0, 1, and 3 dpi (*n* = 3 biological replicates) and statistically compared with WT control strain using student’s *t*-tests (*P < 0.05, ns non-significance). (**F**) To verify the successful mutation of gene *C_22735* (*tetR1*), the fold change relative expression levels were detected and compared in the tested strain S26, Δ22735, C22735, and OE22735. qRT-PCR-based fold change expression of the gene of interest *C_22735* (*tetR1*) was recorded with reference gene *gyrB*. Analysis of variance (ANOVA) was employed (*n* = 3 biological replicates), and different letters indicate significant differences at *P* < 0.05. (**G**) S26, S26-Δ22735, S26-C22735, and S26-OE22735 were individually injected into *N. benthamiana* leaves. Leaves were stained with trypan-blue dye after 36 h, and the initial bacterial concentration was 5 × 10^7^ CFU/mL (*n* = 3 leaves per dilution). (**H**) The expression of *C_22735* (*tetR1*) was detected by western blot using the Myc.A7. M: standard protein marker. Lanes 1–3 represent the overexpressed strain OE22735, lanes 4–6 represent the complementary strain C22735, and lanes 7–9 represent the empty control strain.

To assess the role of the TetR/AcrR transcription factor TetR1 in Psa pathogenicity, we compared the virulence of wild-type (WT) S26, the Δ22735 (TetR1 knockout), C22735 (complemented), and OE22735 (overexpression) strains. Using kiwifruit leaf discs and branches inoculated with each strain (10⁴ CFU/mL), we observed that Δ22735 exhibited significantly reduced pathogenicity compared to wild-type S26 (Fig. 1A and B). In contrast, both the complemented (C22735) and overexpression (OE22735) strains showed virulence levels comparable to the wild type (Fig. 1A through D). These results demonstrate that TetR1 is essential for full Psa pathogenicity, likely through its regulatory influence on T3SS-mediated virulence mechanisms. To investigate whether this mutation affected the growth of the knock strain, we recovered the CFU until 3 d post-infection (dpi). After 3 dpi, it was significantly lower for Δ22735 compared to the wild-type S26 strain (Fig. 1E). Quantitative reverse transcription PCR (qRT-PCR) analysis was used to confirm the *tetR1* gene expression, which was almost undetectable due to in-frame deletion in the Δ22735 strain, while it was about eightfold higher in C22735 and approximately 70-fold higher in OE22735 relative to the wild type (Fig. 1F). It also confirmed that the deletion of *tetR1* was quite successful and showed the same effect as the Tn mutant revealed on T3SS-mediated pathogenicity.

The T3SS is a key factor in the virulence of Psa, often triggering HR in nonhost plants. To investigate the impact of knocking out the *tetR1* gene on HR induction in the non-host plant *N. benthamiana*, indicated strains were separately injected into *N. benthamiana* leaves, and leaf necrosis was observed after staining with trypan-blue dye after 24 h. The Δ*22735* knockout exhibited a reduced HR compared to S26 control, while the complementary strain C22735 restored HR activity; however, OE22735 did not (Fig. 1G). Western blot analysis confirmed normal expression of TetR1 protein in both the C22735 and OE22735 strains (Fig. 1H). The impaired pathogenicity and reduced HR induction observed with the *tetR1* mutation, alongside phenotype restoration in C22735, indicate that *tetR1* positively regulates T3SS expression. Additionally, the reduced CFUs in the *tetR1* mutant suggest a role for *tetR1* in bacterial survival. It would be intriguing to know how it influences the other genes essential in the T3SS cascade, especially *hrpRS* and *hrpL*.

**Fig 2:**
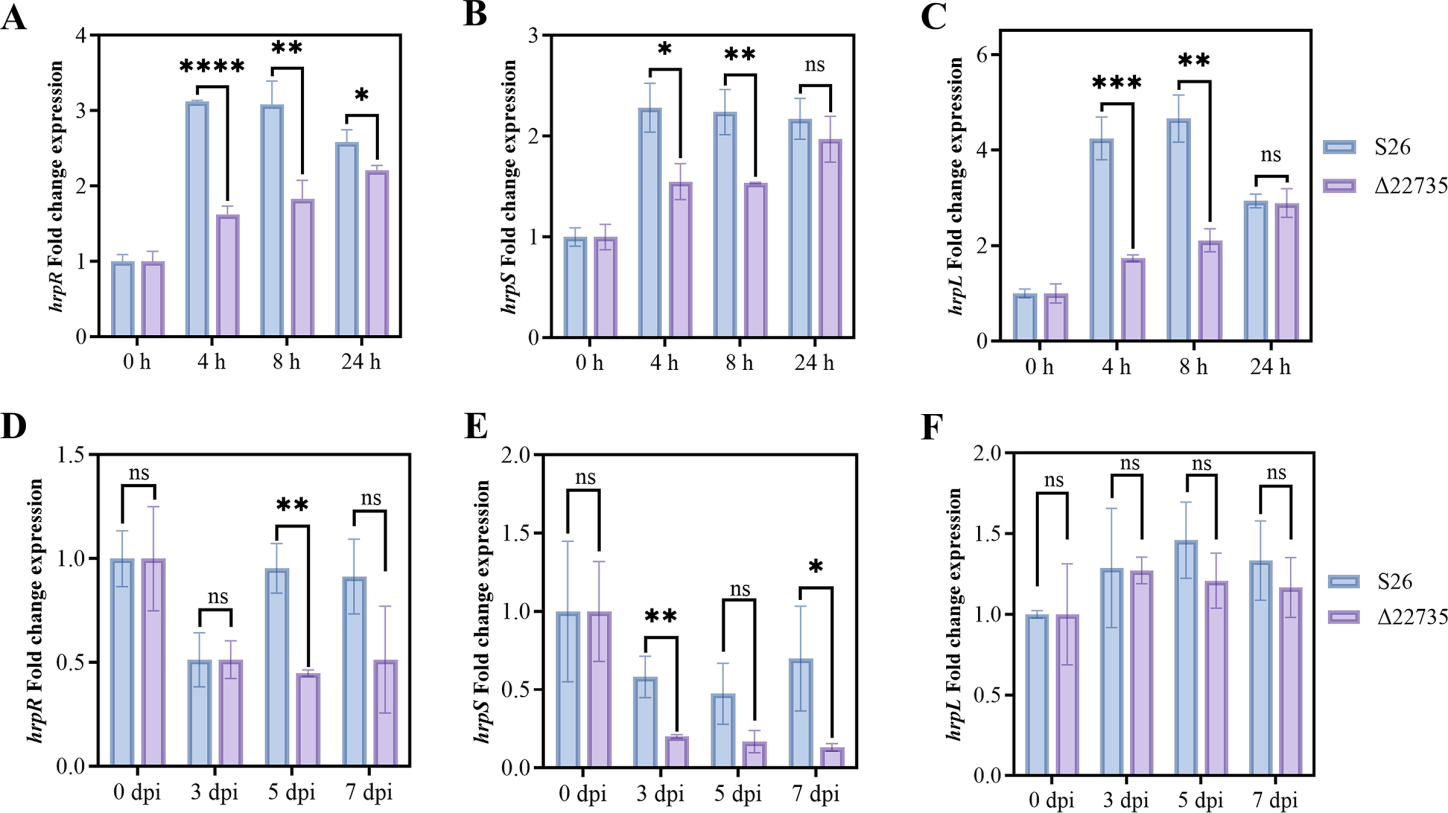
The transcription factor *C_22735* (*tetR1*) is involved in the regulation of T3SS expression in Psa both *in vivo* and *in vitro*. (**A–C**) *In vitro*, qRT-PCR-based fold change expression of the gene of interest *hrpR*, *hrpS*, and *hrpL* was recorded with reference gene *gyrB*. S26 WT and knockout strain Δ22735 (*tetR1* knockout) cells (1.0 × 10^8^) were equally inoculated in hrp-derepressing medium (HDM) fr T3SS induction, and samples were taken at 0 h, 4 h, 8 h, and 24 h to detect the transcript levels of *hrpR*, *hrpS*, and *hrpL*. (**D–F**) S26 WT and knockout strain Δ22735 were inoculated (equal inoculum of 1.0 × 10^8^ cells) on the kiwifruit branches of “Hongyang,” and samples were taken at 0 d, 3 d, 5 d, and 7 d for *in vivo* detection of fold change relative expression of *hrpR*, *hrpS*, and *hrpL*. Student’s *t*-test was used to compare the level of significant differences (ns, no significant difference; *P < 0.05, **P < 0.01, ***P < 0.001, and ****P < 0.0001).

During *in vitro* experiments using qRT-PCR, we dissected the expression levels of T3SS cascade downstream genes *hrpR*, *hrpS*, and *hrpL* in wild-type strain S26 (control) and the *tetR1* knockout strain in hrp-derepressing medium (HDM). The results indicated that mutation in the *tetR1* gene significantly repressed the relative fold changes in expression levels of *hrpR*, *hrpS*, and *hrpL* compared to those in the wild-type strain at both 4 h and 8 h (Fig. 2A through C). During this time-course experiment, the *hrpR* gene was significantly repressed until 24 h, while *hrpS* and *hrpL* showed markedly reduced expression in Δ22735 for 4 h and 8 h compared to the wild-type strain points post-infection, with *hrpL* exhibiting a downward trend, albeit not statistically significant (Fig. 2A through C). Furthermore, the total RNA of the plant was extracted, and bacterial RNA was purified to prepare the first strand cDNA synthesis using commercial kits for qRT-PCR using gene-specific primers. The analysis of obtained results at various time points for 0-7 d post-infection showed that *hrpR* and *hrpS* expression in the *tetR1* mutant was markedly reduced compared to the wild-type strain, with *hrpL* exhibiting a recovered expression trend, albeit statistically not significant (Fig. 2D through F). The observed loss of T3SS activation in the *tetR1* mutant—due to the lack of functional TetR1 protein—and its restoration in the complemented strain, along with the comparison of *hrpR*, *hrpS*, and *hrpL* expression levels, suggest that the native promoter of the *tetR1* gene plays a crucial and indispensable role in the positive regulation of T3SS.

### TetR1 directly binds to the promoter of *hrpR* and *hrpL*

**Fig 3:**
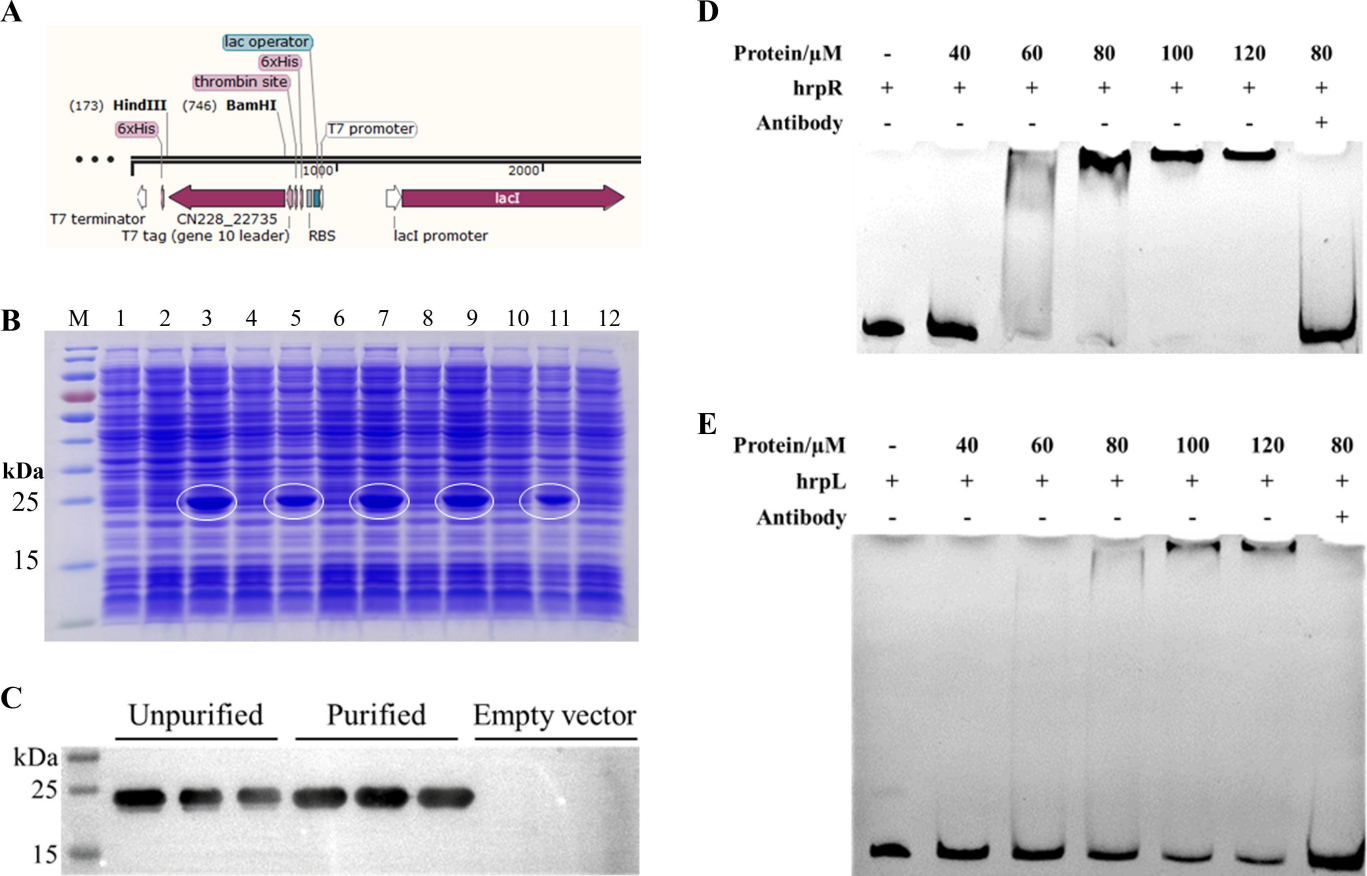
*C_22735* (*tetR1*) could directly bind to the upstream DNA sequences of *hrpR* and *hrpL*. (**A**) Plasmid construct of prokaryotic expression vector pET28a-22735-His. (**B**) SDS-PAGE was used to detect the expression of *C_22735* (*tetR1*) induced by different IPTG concentrations in *Escherichia coli* BL21. Lanes 1, 3, 5, 7, 9, and 11 were the total protein induced by 0 mM, 0.2 mM, 0.4 mM, 0.6 mM, 0.8 mM, and 1.0 mM of IPTG, respectively. Lanes 2, 4, 6, 8, 10, and 12 were empty vectors added with different concentrations of IPTG-induced expression of total protein. The C-terminus fusion protein *C_22735* (*tetR1*) with 6 × His tag was purified and expressed by Ni-NTA preloading column. (**C**) Western blot was used to detect the expression of *C_22735* (*tetR1*) in *E. coli* BL21 using rabbit polyclonal antibody against 6 × His. (**D and E**) EMSA showed that C_22735 could directly bind to the non-coding region upstream of *hrpR* and *hrpL* genes. DNA probe position was detected using a nucleic acid dye. The “antibody” in the EMSA experiment is Myc.A7.

To confirm the direct binding of *tetR1* to the upstream non-coding regions of the *hrpR* and *hrpL* genes, we constructed the prokaryotic expression vector pET28a-22735-His (Fig. 3A). Following induction with 0.6 mM isopropyl β-d-1-thiogalactopyranoside (IPTG), *tetR1* (6 × His tag) was purified using a Ni-NTA column (Fig. 3B) and verified by western blot (Fig. 3C). Electrophoretic mobility shift assays (EMSA) were then conducted using DNA probes from the upstream regions of *hrpR* and *hrpL. tetR1* specifically bound each probe, with binding intensity increasing in relation to probe concentration (Fig. 3D and E). The incorporation of the specific antibody led to an increase in the mass of the protein-DNA complex, thereby impeding its entry into the electrophoretic lane, which further verified the specific binding between the protein and DNA. These results demonstrate that TetR1 directly binds to the upstream non-coding regions of *hrpR* and *hrpL*, suggesting its regulatory role in T3SS gene expression.

### TetR1-dependent T3SS expression is influenced by exogenous signals and carbon sources

**Fig 4:**
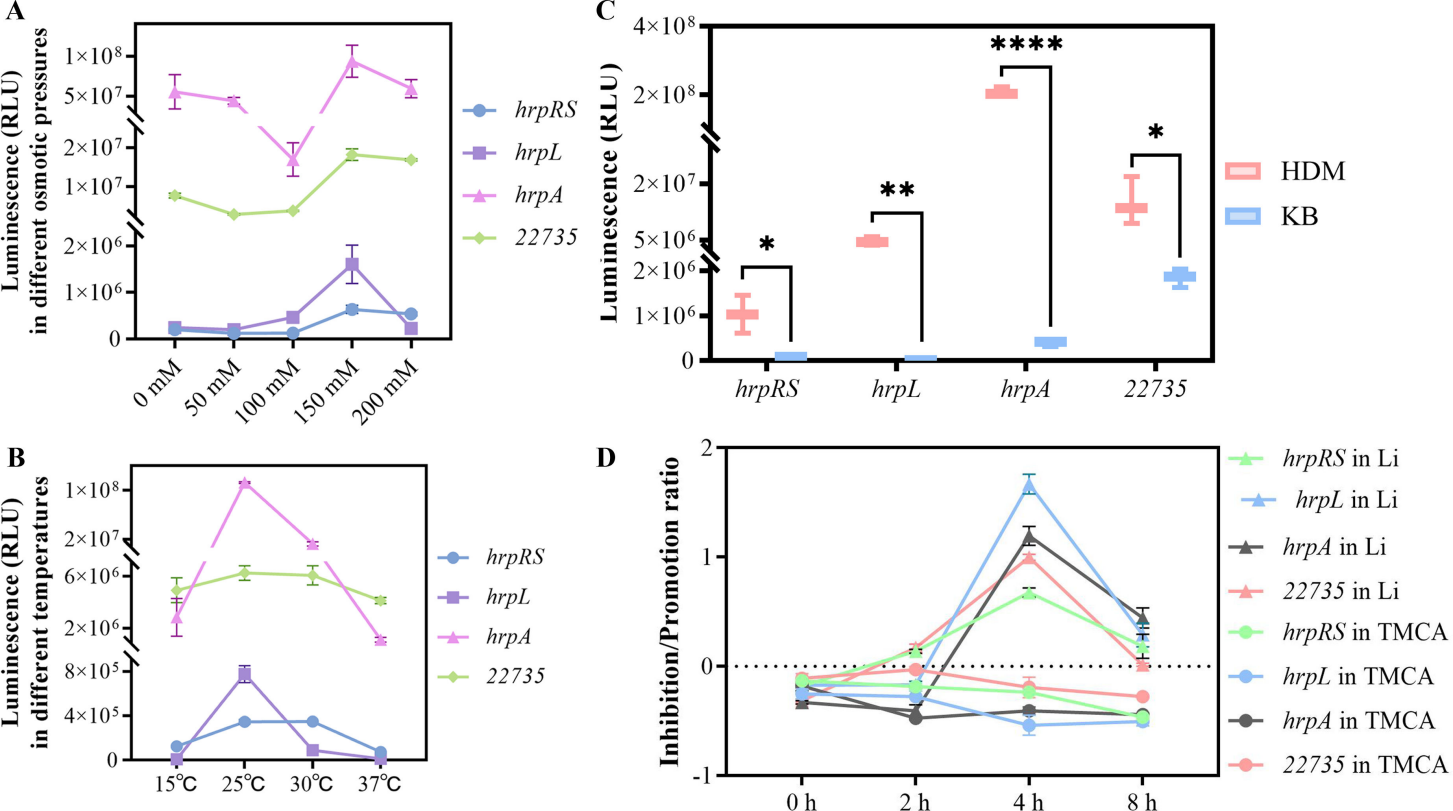
TetR1-dependent T3SS expression is influenced by exogenous signals and carbon sources. (**A**) Different osmotic pressures affected the promoter activities of *C_22735* (*tetR1*) and downstream genes such as *hrpRS*, *hrpL*, and *hrpA*. Each strain was inoculated into HDM supplemented with different concentrations of NaCl (simulating different osmotic pressure environments), and the luciferase expression was detected after 8 h of culture at 25°C. (**B**) Different temperatures affected the promoter activities of *hrpL* and *hrpA*, but not *C_22735* and *hrpR/S*. Each strain was inoculated in HDM and incubated for 8 h at different temperatures to detect the luciferase expression. (**C**) Different nutrient conditions had different effects on the promoter activities of *tetR1* and downstream genes such as *hrpRS*, *hrpL*, and *hrpA*. Minimal media (HDM) activated T3SS, but rich media KB did not. Each strain was inoculated in KB and HDM media and cultured at 25°C for 24 h to detect luciferase expression. (**D**) 4-methoxycinnamic acid (TMCA) and lignin (Li) differentially affected the promoter activities of C_22735 (*tetR1*) and its downstream genes *hrpRS*, *hrpL*, and *hrpA*. Each strain was inoculated in HDM supplemented with either 100 µM TMCA (36) or 100 µM Li, with concentrations adjusted in the culture media at 25°C. Luciferase expression was measured at 0 h, 2 h, 4 h, and 8 h (36) and 100 µM Li, net concentration adjusted in culture media. The luciferase expression was detected after 0 h, 2 h, 4 h, and 8 h. Student’s *t*-test was used to compare the significance of the differences, and S26 served as a control in comparisons (ns, no significant difference; *P < 0.05, **P < 0.01, and ****P < 0.0001). NaCl and carbon sources (10 mM) of each chemical were dissolved in water. In addition, a 100 µM concentration of TMCA and Li was prepared in dimethyl sulfoxide and tested. The fluorescence was detected by luminometer.

To explore external signals influencing *tetR1*-mediated T3SS expression, we constructed nano-luciferase reporter plasmids to examine the promoter activities of *tetR1*, *hrpR/S*, *hrpL*, and *hrpA* under varying conditions. Osmotic stress was simulated by supplementing HDM with different NaCl concentrations, revealing that promoter activity (luciferase expression) for *tetR1* and downstream T3SS genes peaked at 150 mM NaCl (Fig. 4A). Temperature fluctuations specifically affected *hrpL* and *hrpA* expression, with peak activity at 25°C, while *tetR1* and *hrpR/S* maintained stable activity between 25°C and 30°C (Fig. 4B). Furthermore, culturing in the minimal HDM elevated *tetR1* and T3SS gene activities compared to the nutrient-rich King's broth (KB) medium, suggesting that nutrient limitation induces T3SS expression via *tetR1* activation (Fig. 4C). Additionally, we investigated the effects of two agents—4-methoxycinnamic acid (TMCA) (36), which inhibits T3SS expression, and lignin (Li), which promotes it—on *C_22735* transcription in Psa. TMCA suppressed the *tetR1* gene promoter activity, along with that of downstream genes *hrpR/S*, *hrpL*, and *hrpA*, with the strongest inhibition observed at 8 h. In contrast, Li enhanced *tetR1* promoter activity and the expression of *hrpR/S*, *hrpL*, and *hrpA*, peaking at 4 h (Fig. 4D). These findings indicate that TMCA and lignin differentially regulate T3SS expression in Psa by modulating *tetR1* gene transcription.

**Fig 5:**
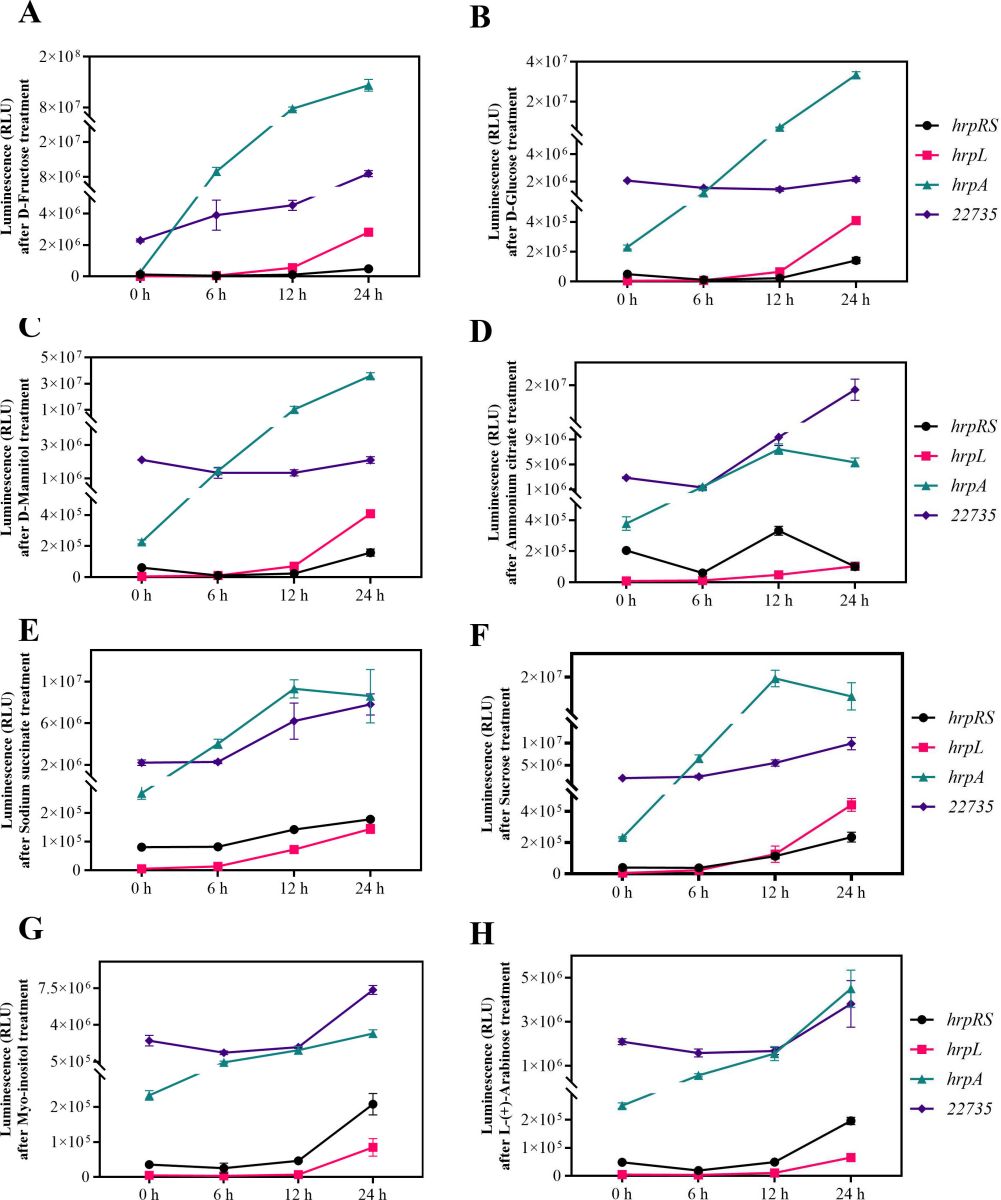
Effects of different carbon sources on the expression of C_22735 (*tetR1*) and T3SS-related genes. Effects of (**A**) D-fructose, (**B**) D-glucose, (**C**) D-mannitol, (**D**) ammonium citrate, (**E**) sodium succinate, (**F**) sucrose, (**G**) myo-inositol, and (**H**) L-(+)-arabinose on the promoter activities of C_22735(*tetR1*), *hrpR/S*, *hrpL*, and *hrpA* genes. The strains were inoculated in HDM containing 10 mM different carbon sources and cultured at 25°C. The luciferase expression was detected after 0 h, 6 h, 12 h, and 24 h.

During further investigation, the impact of various carbon sources—D-fructose, D-glucose, D-mannitol, ammonium citrate, sodium succinate, sucrose, inositol, and L-(+)-arabinose—on the promoter activity of *tetR1* was tested. All compounds, apart from D-glucose and D-mannitol, were found to influence *tetR1* promoter activity (Fig. 5B and C). The effects of these carbon sources on the promoter activities of *hrpR/S*, *hrpL*, and *hrpA* were also assessed, which revealed that the induction of T3SS by D-fructose, L-(+)-arabinose, sodium succinate, sucrose, and inositol may be linked to the elicitation of *tetR1* gene expression, whereas the induction of T3SS by D-glucose (Fig. 5B) and D-mannitol (Fig. 5C) did not correlate with *tetR1* gene.

### The hybrid histidine kinase RetS positively regulates *tetR1* transcription, and transcriptomic analysis reveals shared regulatory pathways

Coincidentally, from the same Tn screening, we identified another candidate gene, *retS*, encoding a hybrid histidine kinase RetS TCS, which reflected similar interaction with *hrpR* and *hrpL* (data not shown). In addition to the Tn mutant, an in-frame deletion mutant of *retS* was constructed in the S26 strain for comparison with *tetR1*. Intriguingly, both *tetR1 mutant* and *retS* knockout strains were infiltrated into kiwifruit leaf discs via immersion methods. Pathogenicity assays revealed that both strains were unable to cause disease in the host plant (Fig. S4A). To gain insights into HR assays, both *tetR1* and *retS* knockout strains were diluted to a 5× gradient along with S26 WT positive control infiltrated to *N. benthamiana* leaves, and HR was observed using trypan blue staining. Buffer was used as a negative control. Similar to that of the *tetR1* mutant strain, the *retS* knockout strain displayed reduced HR induction compared to the positive control strain exhibiting induced HR activity (Fig. S4B). Statistical analysis of recorded disease incidence areas confirmed these results when compared to the S26 control strain or water control (Fig. S4C). It suggested that both knockout strains show significantly impaired pathogenicity on the host plant and reduced HR on the non-host plant, highlighting the crucial regulatory effect in T3SS regulation.

To monitor the effect of *tetR1* and *retS* gene mutations on downstream T3SS regulatory genes *hrpRS*, *hrpL*, and *hrpA*, the S26 control strain was transformed with luciferase reporter plasmids pHrpRS, pHrpL, and pHrpA, as well as in knock-out strains. The indicated strains containing luciferase reporter were tested *in vivo* in HDM. These tests revealed that T3SS regulatory genes *hrpRS*, *hrpL*, and *hrpA* were significantly repressed in *tetR1* mutant, with the highest luminescence observed in control. Interestingly, *hrpRS* and *hrpA* expression levels significantly recovered in *retS* mutant strain, although *hrpL* induction remained significantly lower than S26 WT (Fig. S4D). These results suggested that TetR1 might serve as the response regulator of the hybrid histidine kinase RetS to regulate T3SS and virulence by repressing *hrpRS* and *hrpL*, with no direct effect on *hrpA* gene.

To dissect the regulatory network governing T3SS, we performed RNA-seq on *Psa* wild type (S26), *tetR1* mutant, *retS* mutant, and a T3SS-deficient hrpR* strain (carrying a promoter mutation that abolishes *hrpR* expression). The hrpR* strain’s transcriptome enabled us to point out the substantial role of *tetR1* toward *retS* in a similar type of action during infection.

**Fig 6:**
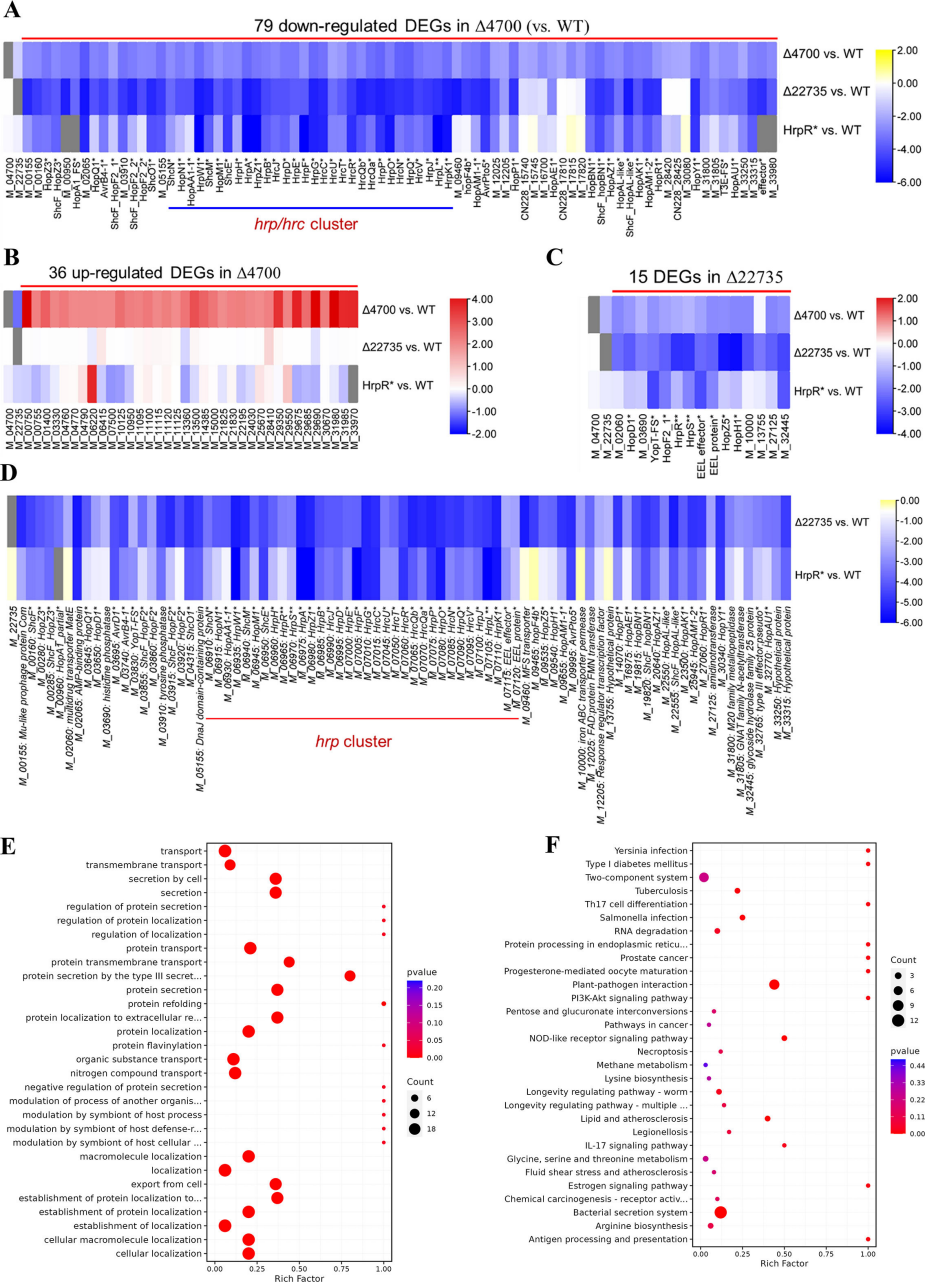
The hybrid histidine kinase RetS positively regulates *tetR1* transcription. (**A–C**) RNA-seq was performed after WT strain S26 and gene knockout strain Δ4700 were cultured in HDM for 8 h, with three biological replicates in each group. A total of 115 DEGs were screened with the threshold of “|log2FoldChange| > 2.0 and false discovery rate (FDR) < 0.01.” (A) Downregulated DEGs. (B) Upregulated DEGs. (C) Fifteen other downregulated DEGs in Δ22735 did not intersect with Δ4700 downregulated DEGs. (**D**) Using |log2FoldChange| > 2.0 and FDR < 0.01 as the threshold, 83 DEGs were screened. (**E and F**) GO biological process functional enrichment analysis (**E**) and KEGG functional enrichment analysis (**F**) of differential genes were performed using ClusterProfiler, and the picture only shows the top 30 enrichment results with lower *P* values.

The *retS* mutant exhibited 115 differentially expressed genes (DEGs: 79 downregulated and 36 upregulated), including 56 T3SS-associated genes (*hrpL*, *hrpA*, effectors, and chaperones; Fig. 6A) compared to the S26 control strain. In the *tetR1* mutant, 83 genes were downregulated, with 68 overlapping with *retS* mutant compared to the S26 control strain (Fig. 6B). This striking overlap suggests RetS and TetR1 co-regulate a core T3SS regulon. *tetR1* expression was significantly reduced in *retS* mutant (log2FC = −1.14), while *retS* expression remained unchanged in *tetR1* mutant (Fig. 6C). This unidirectional relationship positions TetR1 as a downstream activator of RetS, which in turn drives T3SS gene expression. It can be inferred that TetR1 possibly serves as the response regulator of RetS since it is a hybrid histidine kinase TCS. However, there is no direct evidence supporting the interaction between RetS and TetR1 beyond this transcriptome, necessitating further studies for validation.

In *tetR1* mutant, downregulated genes were enriched for T3SS components (e.g., *hrpR*, *hrpS*, *hrpL*, structural genes, and effectors; Fig. 6D), mirroring the transcriptional profile of the T3SS-deficient hrpR* strain. Functional enrichment (Gene Ontology [GO]/Kyoto Encyclopedia of Genes and Genomes [KEGG]) highlighted T3SS secretion, TCS, T3SS protein transport, and plant-pathogen interaction pathways as primary targets of TetR1 (Fig. 6E and F). These findings suggest that TetR1 predominantly regulates T3SS-related genes, with additional effects on transporters.

## DISCUSSION

This study combines genetic, biochemical, and transcriptomic approaches to demonstrate that *tetR1* positively modulates the transcriptional expression of the T3SS cascade in Psa. This family of transcriptional regulators, present in bacteria, archaea, and fungi (25, 37), is characterized as dual-domain proteins playing crucial roles in diverse cellular activities, including responses to osmotic stress and virulence (37, 38). However, their regulatory mechanisms in Psa remain unclear. We identified a TetR/AcrR transcription factor TetR1 with high conservation at the N-terminus of the gene, suggesting a key role in survival, potentially through regulating virulence or stress-response pathways, and its involvement in core functions. In *P. syringae*, several TetR/AcrR family transcription factors have been annotated and studied. For instance, AefR regulates quorum-sensing and efflux genes in pv. *tabaci* 11528 and pv. *tomato* DC3000 (39), while PsrA controls RpoS production, indirectly influencing T3SS via *hrpL* expression in DC3000 (40). Given *tetR1*’s conservation, understanding its regulation could provide opportunities for developing targeted treatments or genetic interventions, particularly by targeting its variable ligand recognition domain, but this requires further studies.

The *tetR1* mutant exhibited diminished pathogenicity in kiwifruit and impaired HR in *N. benthamiana*, both restored by complementation (C22735). This underscores *tetR1*’s role as a positive regulator of T3SS, which is critical for host-pathogen interactions. The reduced expression of *hrpA* (a T3SS structural gene) and the T3SS regulatory cascade (*hrpRS* and *hrpL*) in *tetR1* mutant further supports its upstream regulatory role. Notably, the overexpression strain (OE22735) failed to restore pathogenicity, suggesting that optimal T3SS activation requires tightly regulated TetR1 levels with its native promoter, while its overexpression may disrupt transcriptional balance, potentially by saturating binding sites or interfering with downstream interactions. EMSA confirmed direct binding of TetR1 to the promoters of *hrpR* and *hrpL*, positioning it as a transcriptional activator within the T3SS cascade. Reduced *hrpR/S/L* expression in *tetR1* mutant during early infection aligns with the need for T3SS activation during host colonization. The reduction in *hrpR* and *hrpS* expression *in vivo* and *in vitro*, and a downward trend in *hrpL*, suggests that *tetR1* enables T3SS activation during initial infection phases, critical for bacterial establishment. A parallel example is the auxin response regulator, *aefR*, a TetR family repressor regulating virulence in *P. syringae* pv. *tomato* DC3000 (41). While TetR regulators often control efflux pumps (42, 43), *tetR1*’s link to T3SS highlights its unique role in virulence. However, the complexity of T3SS regulation involving enhancer-binding proteins like HrpR/S (19), implies that *tetR1* is one component of a broader network. However, knocking out a single factor like TetR1 protein may not fully capture the intricate T3SS regulatory network. We propose future studies identify and mutagenize specific binding motifs for *tetR1* within *hrpR*, *hrpS*, and *hrpL* promoters, generating site-specific mutants for a precise dissection of its role, confirming our findings and deepening insights into Psa’s virulence regulation.

Moreover, the precise interpretation of environmental signals is critical for virulence regulation, with factors such as temperature, pH, osmolality, carbon sources, and host-secreted signals influencing T3SS-mediated invasion in Psa (44–46). Our study highlights how environmental factors influence *tetR1*’s regulation. Promoter activity of *tetR1* and T3SS genes peaked in HDM with 150 mM NaCl, suggesting osmotic pressure enhances T3SS-related virulence under host-like conditions, as aligned with another study describing regulatory proteins acting as receptors for small molecule signals or morphogens (47). Temperature influenced *hrpL* and *hrpA* expression independently of *tetR1*, indicating T3SS components adapt to temperature shifts in fluctuating host environments. Higher activity in minimal HDM vs nutrient-rich KB medium reflects nutrient-limited conditions activating T3SS pathways within host tissues. Interestingly, D-fructose and inositol induced *tetR1* expression, while D-glucose and D-mannitol did not, suggesting alternative regulatory pathways. The chemical agents TMCA and lignin further modulate *tetR1* transcription, highlighting environmental signal integration in T3SS regulation. This study aligns with the report of aspartic acid and glutamic acid in leaf tissues, serving as a potent *hrp*-inducing amino acid (48) and acting as chemotactic signals for DC3000, linking nutrient sensing, motility, and T3SS deployment (49).

The *retS* mutant strain exhibited phenotypic effects similar to the *tetR1* mutant, with both mutants showing significantly reduced T3SS gene expression (e.g., *hrpL* and *hrpA*) and attenuated pathogenicity in kiwifruit. The identification of *retS*, a hybrid histidine kinase TCS, as an upstream regulator of *tetR1*, places it critically within the T3SS pathway. *retS* knockouts, like *tetR1* mutant, show significant T3SS gene downregulation, indicating RetS upregulates *tetR1*, transducing signals to initiate T3SS activity during infection. The identified *retS* is totally different from those already studied in *P. syringae* pv. syringae B728a or exemplified as RetS and LadS sensor kinases of the human pathogen *P. aeruginosa*. We identified this hierarchical relationship for the first time, which positions *retS* as a signal sensor and *tetR1* as a transcriptional effector. Overlapping downregulated genes (e.g., *hrpL* and *hrpA*) suggest tight coordination, while *retS* mutant-specific upregulated genes imply additional regulatory roles for *retS*. Further investigation into *tetR1* interactors could unravel cross-regulatory networks governing T3SS activation. None of the 36 upregulated genes in *retS* mutant were differentially expressed in *tetR1* mutant, implying *retS*’s additional regulatory functions, revealing bacterial adaptability. Identifying other transcription factors interacting with *tetR1* could deepen the understanding of T3SS activation and pathogenesis. Thus, *tetR1*’s role in T3SS regulation, its conservation, and its environmental sensitivity underscore its importance in Psa virulence, offering avenues for future research and potential disease control strategies.

### Conclusions

This study demonstrates that *tetR1* is a critical regulator of the T3SS cascade in Psa, orchestrating key pathogenicity processes. Through a combination of genetic, biochemical, and transcriptomic approaches, we established that *tetR1* positively modulates the expression of the *hrpR*/*S* and *hrpL* T3SS pathway by directly binding to the upstream regulatory regions of *hrpR* and *hrpL* genes. Mutation in *tetR1* led to significant reductions in T3SS gene expression and diminished pathogenicity in kiwifruit, while complementation restored these traits, underscoring its essential role *in vivo* and *in vitro*. Additionally, identified *retS* acts as an upstream regulatory component, further suggesting that *tetR1* functions within a coordinated regulatory network, influenced by this TCS to drive T3SS activation. We further found that the presence of carbon sources such as fructose and sucrose, along with chemicals like lignin and TMCA, influenced T3SS, indicating that *tetR1*’s regulatory activity is responsive to environmental cues that Psa might encounter *in planta*. Enrichment analysis also highlights that *tetR1* likely plays a focused role in modulating T3SS-related pathways, reinforcing its significance in Psa’s virulence mechanism. Together, these findings contribute to our understanding of T3SS regulation in plant-pathogenic bacteria and offer a foundation for future strategies to mitigate Psa-related crop losses.

## MATERIALS AND METHODS

### Strains, plasmids, and growth conditions

Bacterial strains, plasmids, and primers used in this study are listed in Table S1. *Pseudomonas syringae* pv. *actinidiae* (Psa) strain M228 was a previously published strain (50), which was used for gene knockout and luciferase reporter strain construction. Psa3 strain M228 was referred to as strain S26. Strain S26-pHrpRS:Nluc, S26-pHrpL:Nluc, S26-pHrpA:Nluc, and S26-p22735:Nluc were incubated in Luria-Bertani(LB) medium with shaking at 200 rpm for 16 h. *Escherichia coli* DH5a strain was used to clone gene fragments, and *E. coli* S17-1λpir was used for conjugative transfer. The suicide plasmid pK18mobSacB (51) was used for gene knockout. The expression plasmid pMEKm12 (52) was used to construct the overexpression vector. The expression plasmid pDSK-GFPuv was used for gene complementation and construction of a luciferase reporter in strains. The plasmids pET28a were transfected into the *E. coli* BL21 (DE3) for protein expression in strains. Psa strains and mutants were grown in LB, KB, or HDM at 25°C. *E. coli* strains were cultivated in LB medium at 37°C.

### Construction of the transposon library and screening and identification of mutants

The Psa S26-cHrpA::Nluc strain was cultured overnight at 25°C with shaking at 200 rpm. The strain was activated and cultured overnight at 25°C and 200 rpm in LB. The culture was adjusted to an OD_600_ of 1.0 and inoculated into fresh LB medium at a 1:50 ratio. After growing to mid-log phase, cells were harvested by centrifugation at 1,700 × *g* for 5 min and resuspended in 20 mL of pre-chilled sterile water. The washing and resuspension steps were repeated three times, followed by resuspension in 1 mL of pre-chilled 10% glycerol. Aliquots of 100 µL were stored at −80°C for further use. The reporter strain S26-cHrpA::Nluc competent cells were mixed with 100 ng of the transposon plasmid pSAM-CAM. The mixture was subjected to electroporation using a Gene Pulser II apparatus (Bio-Rad) adjusted to 2.5 kV, 6 ms pulse. After electroporation, cells were recovered in 1 mL of non-selective LB medium at 25°C with shaking at 200 rpm for 1 h. Cells were then plated onto LB agar plates containing 50 µg/mL kanamycin and incubated at 25°C for 48 h to select transformants.

Individual colonies were inoculated into LB medium containing 50 µg/mL kanamycin and cultured overnight at 25°C with shaking at 200 rpm. Cells were harvested by centrifugation at 5,000 rpm for 5 min, resuspended in sterile water, and adjusted to an OD_600_ of 1.0. Each strain was inoculated into a new white 96-well plate containing T3SS induction medium (HDM) at a 1:10 ratio. After 24 h of incubation at 25°C, 100 µL of each culture was transferred to a new 96-well plate, and 20 µL of luciferase substrate was added. Luminescence was measured using a luminometer. Mutants exhibiting significant differences in luminescence compared to the wild-type strain were selected for further analysis.

Selected mutants were tested for swimming motility by inoculating 5 µL of bacterial suspension (OD_600_ = 0.2) onto the center of a 0.3% LB agar plate. After 72 h of incubation at 25°C, the diameter of the swimming zone was measured. Genomic DNA was extracted from mutants with significant differences in luciferase expression levels. Inverse PCR was performed using primers P0F/P6R, hrpANLuc-F/R, and Tn seq-F/R to amplify regions flanking the transposon insertion sites. The PCR products were sequenced, and insertion sites were identified by BLASTN alignment against the Psa S26 reference genome. Genes affected by the insertions were annotated using Annovar software.

### Construction of in-frame deletion mutant of C_22735 and C_04700, and C_22735 complementation and overexpression strains

Deletion mutant was constructed by homologous recombination, based on the suicide plasmid pK18mobSacB, as described earlier (32). Deletion of C_22735 in Psa3-S26-Δ22735 (hereafter referred to as Δ22735) was detected by primers P0F/P6R, SacB-F/R, and 22735RT-F/R (Table S1). Deletion in C_4700 Psa3-S26-Δ4700 (hereafter referred to as Δ4700) was detected by primers P0F/P6R, SacB-F/R, and 4700RT-F/R (Table S1). To generate a complementary (self-promoter) and overexpression strain (Lac promoter) of C_22735, a primer pair 22735ORF-F/R and 22735AP-F/R (Table S1) were used to amplify DNA fragments of 609 bp and 831 bp size from strain S26, respectively (95°C for 15 s, 55°C for 15 s, and 72°C for 15 s). Gel verification was followed by cleaving each fragment and plasmid pDSK-GFPuv using the restriction endonucleases NdeI/BamHI and KpnI/PstI, respectively. Cleaved fragments were purified from gel and ligated using a DNA ligation kit (DNA Ligation Kit Ver.2.1; TaKaRa). Ligated vectors were cloned in DH5α competent cells. PCR- and electrophoresis-mediated verification was carried out using primers GBD-F/R and by Sanger sequencing. The recombinant plasmids were extracted via kit (Plasmid Mini Kit, OMEGA). The complementary strain (referred to as C22735) and overexpression strain (referred to as OE22735) were verified by primers P0F/P6R and M13F/R (Table S1). After sequencing and PCR verifications of strains (Fig. S3), the obtained strains were transformed with pDSK-hrpA-NLuc to monitor T3SS expression.

### Promoter activity assay

To record the promoter activities as luciferase expression, a brief protocol was adapted from references 36, 50. The luciferase reporter plasmid P_hrpRS_-Nluc, P_hrpL_-Nluc, and hrpA-Nluc were electroporated into competent S26, Δ22735, C22735, OE22735, and C_Δ04700 cells, respectively, as constructed previously (Table S1). The bacterial cultures were centrifuged at 5,000 rpm for 5 min, and the pellets were resuspended in sterile water. The bacterial suspensions were adjusted to an OD_600_ value of 1.0 using sterile water. During *in vitro* experiments, each strain was inoculated at a 1:10 ratio into 96-well microplates containing the T3SS-inducing medium HDM and incubated at 25°C for 24 h. Subsequently, 100 µL of the culture from each well was transferred to a new 96-well white microplate. To each well, 20 µL of a mixture of luciferase substrate and buffer (luciferase substrate:buffer = 1:250) was added and mixed thoroughly, and the fluorescence values were measured using a luminometer (GloMax NAVIGATOR, USA). To compare the promoter activities as luciferase expression, the wild S26 strain served as a positive control, and statistical significance was calculated.

### Pathogenicity assessment and recording of bacterial counts

Pathogenicity testing was conducted using established protocols (53, 54). To assess pathogenicity on kiwifruit, test strains S26, Δ22735, OE22735, and/or C_04700 were grown under agitation (200 rpm) at 25°C for 16 h, harvested, washed, and resuspended in sterile water. For leaf disc assays, bacterial suspensions were diluted with sterile water to OD_600_ = 0.2 and supplemented with 0.01% Silwet L-77. Kiwifruit leaves were disinfected with 0.5% sodium hypochlorite for 3 min and washed three times with sterile water. Sterilized kiwifruit leaves were cut into uniform 1.5 × 1.5 cm discs using a 1.5 cm punch. The treated leaf discs were placed into the bacterial suspension and the solution containing only 0.01% Silwet L-77 (blank control), gently shaken to fully contact each leaf disc with the bacterial solution, and then left for 1 h at room temperature. Discs were placed on 0.5% water agar plates in petri plates (90 mm). Lesion areas were measured after 5 d. The Image J software was then utilized to compare the lesion area of each leaf disc. The trimmed twigs (40 cm) of Hongyang were surface-sterilized with 0.5% sodium hypochlorite, rinsed with double distilled water, and air-dried under sterile conditions. Based on twig length, suitable four incisions (2 mm width × 1 mm depth) were made per shoot using a sterile blade. A bacterial suspension (10 µL) was applied for treatment, and sterile water was used for controls to each wound. Twigs were placed in sterile trays containing moist filter paper, while sterile conditions were maintained by wrapping them with plastic sheets. Culture conditions were established as temperature 16°C with a total of 16 h with the interval of 8 h light and dark cycle and 95% humidity level. Lesion development was documented and imaged after 10 d.

Bacterial quantification involved homogenizing three leaf discs per replicate in 1 mL sterile water. Homogenates were serially diluted (10-fold increments), and 0.1 mL aliquots were spread onto LB agar plates using a sterile spreader. After 48 h incubation at 25°C, CFUs were enumerated. The number of bacteria in the original sample was calculated using the following formula:


$$CFU/mL=\frac{Number\;of\;colonies\;counted}{Volume\;of\;sample\;plated\;\left(mL\right)}\times Dilution\;factor$$

where CFU, an abbreviation for CFUs, represents the quantity of viable bacteria.

### Hypersensitivity response assays

A comprehensive protocol was followed from reference 36. Briefly, the test strains (S26 wild type [control], Δ22735, C22735, OE22735, and C_Δ04700) were syringae inoculated in *N. benthamiana* leaves (four leaf stage) to assess the strain’s ability to elicit hypersensitivity in non-hosts. The bacterial suspensions (OD_600_ nm of 0.2) of test strains in 10 mM MgCl_2_ and three bacterial suspensions that were serially diluted fivefold were infiltrated into *N. benthamiana* leaves using a blunt-end plastic syringe, and the diameter of bacterial liquid penetration was about 1 cm. Necrosis of the infiltrated area after 24 h was considered an HR and stained with trypan-blue solution (0.67 mg/mL) for 5 min at 95°C. Then, 2.5 mg/mL chloral hydrate was used for decolorization, and the decolorization solution was replaced every 2 h. Photographs were obtained after sufficient decolorization. S26 wild type served as a positive control, while MgCl_2_ was used as a negative control.

### Protein purification and western blotting

The recombinant plasmid pET28a-22735-Myc was transformed into BL21 competent cells by heat shock and induced with 0.6 mM IPTG at 16°C with significance thresholds set at an adjusted and 200 rpm overnight. Protein isolation and further purification were performed using the previously described protocol (54). The cells were resuspended in 20 mL Binding/Wash Buffer plus phenylmethylsulfonyl fluoride (PMSF) (100 mM). Recombinant proteins were purified with Ni–NTA resin. Purity was assessed by SDS-PAGE, and their concentrations were determined using the Bradford assay (Kruger 1994). The purified protein solution was mixed with sodium dodecyl sulfate (2× SDS) loading buffer, heated at 100°C for 15 min, and loaded for separation by 15% SDS-PAGE along with a page ruler pre-stained protein ladder (Thermo Scientific) for electrophoresis. Proteins were then transferred to 0.45 µm Trans-Blot Turbo Mini PVDF membrane (Bio-Rad). The membrane was blocked with 5% skim milk in tris buffer saline containing 0.1% Tween-20 for 1 h at room temperature and incubated with Myc Tag Monoclonal Antibody (Myc.A7, Invitrogen) overnight at 4°C. The membrane was next incubated with horseradish peroxidase-coupled secondary antibodies for 1 h at room temperature. Protein bands were revealed by the Star Signal Plus Chemiluminescent Assay Kit (Genstar), and chemiluminescence signals were detected by a protein imaging system (Tanon 6600multi, Shanghai, China).

### Electrophoretic mobility shift assay

Biotin-labeled or unlabeled primers CL2F/CL7R and HrpL-F/R (Table S1) were used to amplify 976 bp and 262 bp DNA probe fragments from strain S26, respectively, followed by gel purification. After ligation and cloning in BL21-pET28a-22735-Myc, its cellular nuclear extracts were prepared using B-PER Bacterial Protein Extraction reagent (Thermo Scientific) and purified. Such pure proteins were used for binding reactions performed in the Lightshift EMSA Optimization and Control kit (Thermo Scientific) according to manufacturer instructions. For each reaction, we added 100 ng of the probe. Different amounts of protein were used to assess the binding characteristics. In the super-shift reaction, we added 2 µL of the antibody (Myc.A7, Invitrogen) at a concentration of 1 mg/mL. Blots were detected using the Chemiluminescent Nucleic Acid Detection Module (Thermo Scientific) and visualized with an imaging system (Tanon 6600multi, Shanghai, China).

### RNA extraction and reverse transcription-qPCR

We performed *in vitro* RT-qPCR analyses to evaluate the expression levels of *hrpR/S*, *hrpL*, and *hrpA* in the C_22735 knockout strain and compared them to the wild-type strain. The tested strains S26, Δ22735, and OE22735 were cultured at 25°C and 200 rpm for 16 h, centrifuged at 5,000 rpm for 5 min, washed with sterile water three times, and suspended in 200 µL of sterile water. The bacterial suspension was added to 1 mL of HDM (T3SS inducing condition) at a ratio of 1:50 and cultured at 25°C for 6 h. For *in vivo* analysis, wild-type strain S26 and knockout strain Δ22735 were resuspended in LB medium at 25°C, 200 rpm/min to logarithmic growth phase, 5,000 rpm/min for 5 min, and then in sterile water to OD_600_ = 1.0. A 20 cm kiwifruit branch was sterilized with 0.5% sodium hypochlorite for 15 min and rinsed three times with sterile water, and four 1–2 mm wounds were cut. Ten microliter of the inoculated cell suspension was applied to the scratch, placed on a tray sealed to moisten, and placed in a 16°C. Samples were collected at 0 dpi, 3 dpi, 5 dpi, and 7 dpi (three replicates/treatment). RNA was extracted using the Plant RNAexr kit (Cat.#0416, huayueyang). Samples were treated with RNA Protect Bacteria Reagent (Cat. #: 76506, QIAGEN, Shanghai, China), and RNA was then extracted with a bacterial RNA extraction kit (Cat. #: K0731, Thermo Fisher Scientific, Shanghai, China). For RNA quantification prior to reverse transcription to cDNA generation, we utilized the NanoDrop to measure the RNA concentration (detection range: 50-100 ng/µL), and an equal amount of RNA (1 µg) was used as the template for reverse transcription (Cat. K1691, Thermo Scientific). Meanwhile, the integrity of RNA was verified by agarose gel electrophoresis (28S/18S ratio >1.8), ensuring that there were no significant differences in RNA quality among different strains. For each sample, three biological replicates and three technical replicates were set up. Additionally, a no-template control and a no-reverse transcriptase control were included to rule out genomic DNA contamination. The SD of ∆Ct values was less than 0.5, indicating good experimental reproducibility. For each T3SS gene, qRT-PCR primers are listed in Table S1. Briefly, *hrpR* primer pair hrpR-RT-F /R to amplify a 213 bp fragment, *hrpS* pair *hrpS*-RT-F /R to amplify a 269 bp fragment, and *hrpL* primer pair *hrpL*-RT-F /R to amplify a 130 bp fragment. The qRT-PCR was performed in the Bio-Rad CFX96 system, with gyrB-RT-F/R as internal reference primers. The total reaction system was 20 µL. The reaction program was 95°C for 10 min, followed by 40 cycles of 95°C for 15 s, 60°C for 15 s, and 95°C for 10 min. The 2^−ΔΔCT^ method was used for data analysis (55).

### Transcriptome sequencing and analysis

The tested strains S26, *C_22735*, and *C_04700* mutants were cultured in LB medium without antibiotics at 25°C and 200 rpm for 16 h. G126 (GenBank accession ID: JBLLLG0) isolated from the kiwifruit branch was used as *hrpR, which was a T3SS-deficient strain. The cultures were washed thrice with sterile water and then suspended with 200 µL sterile water. The suspensions were added to 1 mL HDM (T3SS-induced condition) at a ratio of 1:50 and cultured at 25°C for 6 h. Samples were pretreated with RNA Protect Bacteria Reagent (Qiagen), and total RNA was extracted by the bacterial RNA extraction kit (Cat. K0731, Thermo Scientific) and sent to a sequencing company (MAGIGENE Co., Ltd., Guangdong, China) for transcriptome sequencing. The total RNA was stabilized using RNA Protect Bacteria Reagent (QIAGEN). RNA quality was assayed on a “Bioanalyzer 2100 system” (Agilent Technologies). Commercially available kits were used to prepare the first and second strand cDNA, and the residual activity of RNA was resolved using RNase-H. After all the processes, second-strand cDNA was generated using the cDNA Synthesis Kit (Invitrogen, A48570). Illumina adapters were ligated to synthesize the sequencing libraries to size-selected cDNA fragments (370–420 bp) by AMPure XP system (Beckman Coulter). Paired-end sequencing (150 bp reads) was performed on an Illumina NovaSeq platform by a commercial sequencing provider (MAGIGENE Co., Ltd., Guangdong, China), prior to library quality validation using the Bioanalyzer 2100 system. The obtained raw reads were processed by Trimmomatic v0.36 to remove low-quality bases and adapters. Clean reads were aligned to the reference genome (M228) using Bowtie2 v2.2.3. Gene expression quantification (read counts per gene) was performed with HTSeq v0.6.1, and normalized expression values (fragments per kilobase of transcript per million mapped reads) were calculated. Differential gene expression analysis was conducted using DESeq2 (v1.18.0) with significance thresholds set at an adjusted *P*-value < 0.05. GO enrichment and KEGG pathway analyses of DEGs were performed using GOseq (R package) and KOBAS v2.0 (56), respectively. For genome annotations, reference genome M228 (accession ID: CP032631.1) was used to map the T3SS genes and the other pathways.

### Application of exogenous signals (carbon sources and chemical and media conditions)

The test strains S26-pHrpRS:Nluc, S26-pHrpL:Nluc, S26-pHrpA:Nluc, and S26-p22735:Nluc were incubated in LB medium, and collected cells were added to 96-well plates containing HDM (10:1; HDM: bacterial suspension). Different nutritional conditions (HDM and KB), osmolarity (0 mM, 50 mM, 100 mM, 150 mM, and 200 mM NaCl), and carbon sources (10 mM of each chemical like D-fructose, D-glucose, D-mannitol, diammonium citrate, sodium succinate, sucrose, inositol, and L-(+)-arabinose) were prepared in water. In addition, a 100 µM concentration of 4-methoxycinnamic acid and lignin was prepared in dimethyl sulfoxide and tested. The fluorescence value was detected by luminometer (GloMax, USA).

### Statistical analysis

Software SPSS 19.0 (IBM, Armonk, NY, USA) was used for data analysis and GraphPad Prism 9.2.0 for graphing.

## Data Availability

Transcriptome data were deposited in Gene Expression Omnibus (GEO) of NCBI under the accession ID GSE286487. All other data are made available in the paper. Additional supporting data are present as supplemental files.

## SUPPLEMENTAL MATERIAL

Fig. S1 aem.00494-25-s0001.tif The transcription factor C22735 is widely distributed in the Pseudomonas syringae species complex.

Fig. S2 aem.00494-25-s0002.tif Sequence alignment and Ka/Ks analysis of transcription factor C22735.

Fig. S3 aem.00494-25-s0003.tif PCR electrophoresis detection map of Δ22735, C22735, and OE22735.

Fig. S4 aem.00494-25-s0004.tif Pathogenicity, HR, and promoter activity of genes regulating T3SS cascade.

Supplemental legends aem.00494-25-s0005.docx Legends for Fig. S1 to S4.

Table S1 aem.00494-25-s0006.docx Strains, vectors, and primers used in this study.

ASM does not own the copyrights to Supplemental Material that may be linked to, or accessed through, an article. The authors have granted ASM a non-exclusive, world-wide license to publish the Supplemental Material files. Please contact the corresponding author directly for reuse.
