# Supplementary figures and images for: RetS-mediated environmental sensing coordinates TetR-dependent regulation of type III secretion system and virulence in Pseudomonas syringae pv. actinidiae
Source: Appl Environ Microbiol. 2025 Jun 10;91(7):e00494-25. doi: 10.1128/aem.00494-25 (PMC12285254; doi:10.1128/aem.00494-25)

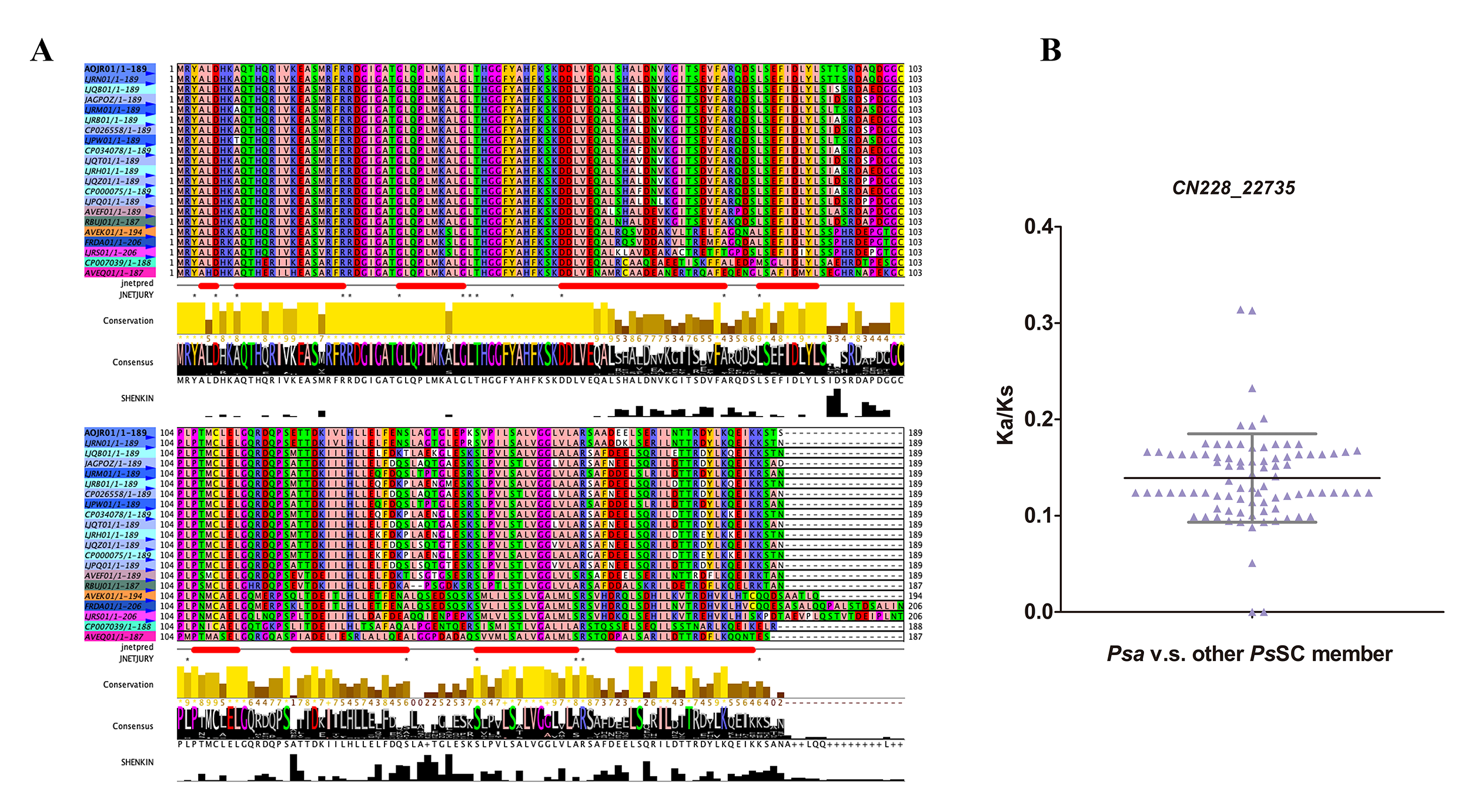

Supplement: Fig. S2 — Sequence alignment and Ka/Ks analysis of transcription factor C22735. [file aem.00494-25-s0002.tif]

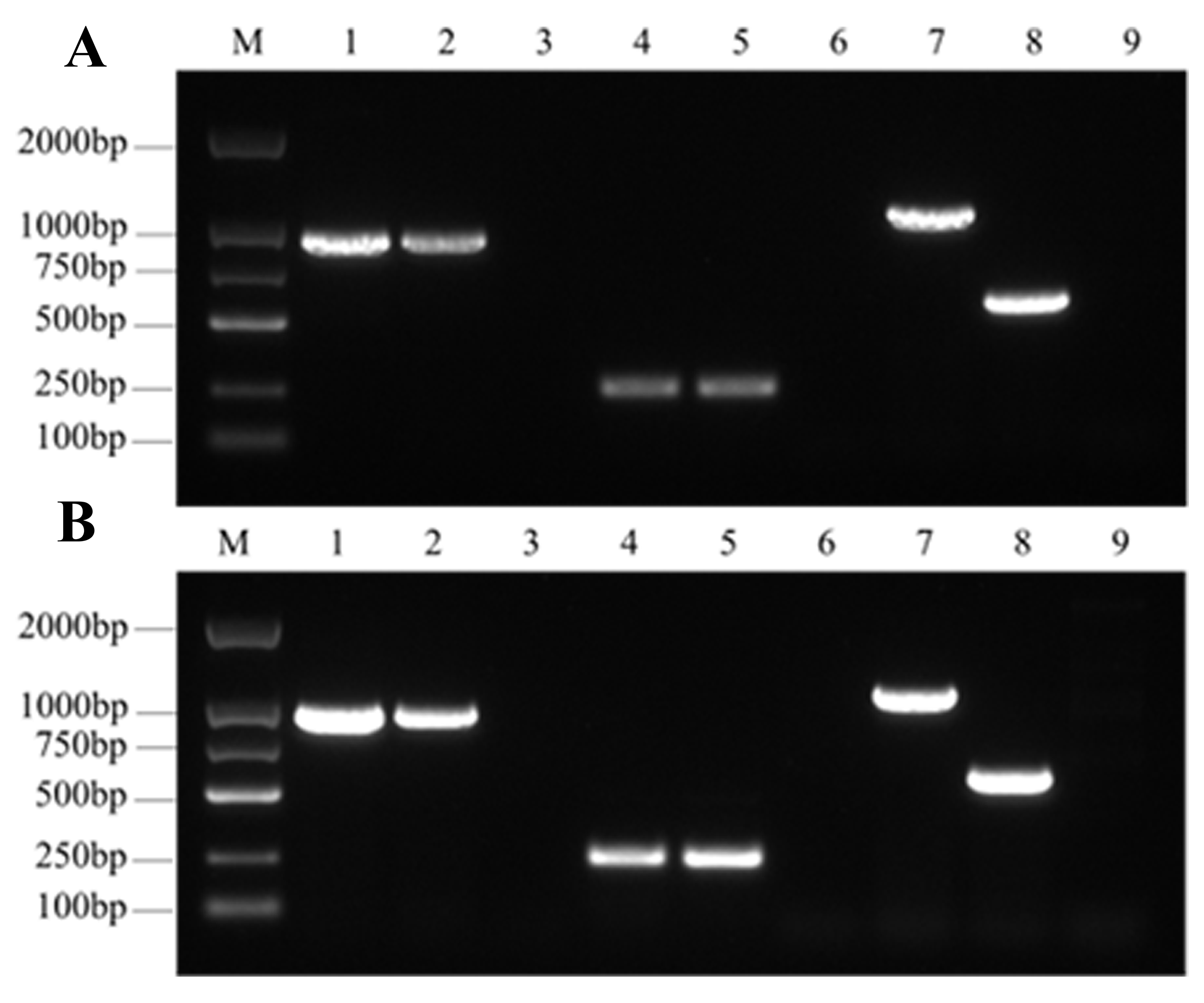

Supplement: Fig. S3 — PCR electrophoresis detection map of Δ22735, C22735, and OE22735. [file aem.00494-25-s0003.tif]
