# Supplementary material for: RetS-mediated environmental sensing coordinates TetR-dependent regulation of type III secretion system and virulence in Pseudomonas syringae pv. actinidiae
Source: Appl Environ Microbiol. 2025 Jun 10;91(7):e00494-25. doi: 10.1128/aem.00494-25 (PMC12285254; doi:10.1128/aem.00494-25)
Supplement: Supplemental legends — Legends for Fig. S1 to S4. [file aem.00494-25-s0005.docx]

**Supplementary Figures**

**Fig. S1** The transcription factor C22735 is widely distributed in the Pseudomonas syringae species complex. Based on the C22735 amino acid sequence, a Bayesian evolutionary tree was constructed. Phylosuit software was used to integrate the modelFinder to search for the optimal model, and an evolutionary tree was constructed using MrBayer software. PG stands for phylogroup, T stands for type strain, and PT stands for Pathotype strain. The green circle at the node indicates a Bayesian posterior probability>0.9.

**Fig. S2** Sequence alignment and Ka/Ks analysis of transcription factor C22735. (A) Analysis of amino acid sequence alignment of C22735. MAFFT software was used for comparison, and JalView software was used for analysis. (B) Kiwifruit ulcer pathogen C22735 and *Pseudomonas syringae* species complex (PsSC), The distribution of Ka/Ks values among other members. TBtools was used to calculate the Ka/Ks value between two factors.

**Fig. S3** PCR electrophoresis detection map of Δ22735, C22735 and OE22735. Lane M DNA standard marker (DL2000). Lanes 1 and 2 in each graph are the electrophoresis bands amplified from the positive control and the strain using primers M13F/R, with sizes of 921 bp/949 bp, respectively. Lanes 4 and 5 are the electrophoresis bands of the wild-type strain S26, and the strain is amplified using primers P0F/P6R, with a size of 243 bp. Lanes 7 and 8 are the electrophoresis bands of the wild-type strain S26 and the complementation strain amplified using primers 22735KO-F/R, with sizes of approximately 1128 bp and 588 bp, respectively. Lanes 3/6/9 are blank controls.

**Fig. S4** Pathogenicity, HR and promoter activity of genes regulating T3SS cascade. (A) The tested strains Δ22735 and Δ4700 were infiltrated via immersion method on kiwi-fruit leaf discs and necrosis symptoms on leaf disc were recorded after 5 days post-inoculation. Negligible necrotic symptoms on leaf disc disease are evident on both mutant strains Δ22735 and Δ4700. (B) Control strains S26, knockout strain Δ22735, and C_04700 were individually injected into benthi (*Nicotiana benthamiana*) leaves. Leaves were stained with trypan-blue dye after 36 h, and the initial bacterial concentration was 5 × 10^7^ CFU/mL. (C) S26, Δ22735, Δ4700 were individually injected into benthi (*N. benthamiana*) leaves. Leaves were stained with trypan-blue dye after 36 h, and the initial bacterial concentration was 5 × 10^7^ CFU/mL (n = 3 leaves per dilution). HR induction was observed and compared to buffer solution. (D) S26 WT and both knockout strains (Δ22735, Δ4700) cells recovered from LB culture were mixed at a 1:10 ratio into 96-well microplates containing the T3SS-inducing medium HDM medium after adjusting OD600_nm_ to 1.0 and recorded luciferase expression of plasmid borne pDSK-Nluc containing promoters of hrpR, *hrpS, hrpL, hrpA* genes of T3SS to detect their Luminescence as a relative lux units (RLU) with three biological replicates after 24 hour. Student's t test was used to compare the significance of the differences and S26 served as control in comparisons (**P<0.01, ****P<0.0001).
