# Supplementary material for: RetS-mediated environmental sensing coordinates TetR-dependent regulation of type III secretion system and virulence in Pseudomonas syringae pv. actinidiae
Source: Appl Environ Microbiol. 2025 Jun 10;91(7):e00494-25. doi: 10.1128/aem.00494-25 (PMC12285254; doi:10.1128/aem.00494-25)
Supplement: Table S1 — Strains, vectors, and primers used in this study. [file aem.00494-25-s0006.docx]

**List of Tables**

**Table S1 Strains, vectors, and Primers used in this study**

| **Strains** | **Description** | **Reference** |
| --- | --- | --- |
| ***Pseudomonas syringae* pv. *actinidiae*** | | |
| M228 (S26) | Psa3, Wild-type | Zhao et al., 2015 |
| S26-△22735 | Deletion mutant of C_22735 | This study |
| S26-Δ4700 | Deletion mutant of C_04700 | This study |
| S26-△22735-pDSK-c22735 | Complementary strain of C_22735 | This study |
| S26-pDSK-Lac-22735 | Overexpression strain OE22735 | This study |
| S26-P_HrpRS_:Nluc | Detect *hrpRS* gene promoter activity in Wild-type | Xie et al.,2023 |
| S26-P_HrpL_:Nluc | Detect *hrpL* gene promoter activity in Wild-type | Xie et al.,2023 |
| S26-HrpA:Nluc | Detect *hrpA* accumulation in Wild-type | This study |
| S26-P_22735_:Nluc | Detect C_22735 gene promoter activity in Wild-type | This study |
| △22735-P_HrpRS_:Nluc | Detect *hrpRS* gene promoter activity in deletion mutant | This study |
| △22735-P_HrpL_:Nluc | Detect *hrpL* gene promoter activity in deletion mutant | This study |
| △22735-HrpA:Nluc | Detect *hrpA* accumulation in deletion mutant | This study |
| △22735-P_22735_:Nluc | Detect C_22735 gene promoter activity in deletion mutant | This study |
| **Plasmids** | | |
| BL21-pET28a-22735-Myc | Prokaryotic protein expression vector | This study |
| *Escherichia coli* DH5α | Cloning vectors | Lab stock |
| *E. coli* S17-1λpir | Conjugative transformation vector | Lab stock |
| *E. coli* BL21 (DE3) | Prokaryotic expression | Lab stock |
| pDSK-P_HrpRS_:Nluc | Luciferase reporter vector of *hrpRS* gene promoter | This study |
| pDSK-P_HrpL_:Nluc | Luciferase reporter vector of *hrpL* gene promoter | This study |
| pDSK-HrpA:Nluc | Luciferase reporter vector of *hrpA* accumulation | This study |
| pDSK-P_22735_: Nluc | Luciferase reporter vector of *C_22735* gene promoter | This study |
| pDSK-Lac-22735 | overexpression vector of C_22734, Lac-promoter | This study |
| pDSK-22735 | Complementary vector of C_22735, self-promoter | This study |
| pK18mobSacB | Vector for creating knockout strain | (Schafer et al., 1994) |
| pK18-22735 | Homologous recombination vector for C_22735 gene | Tsingke |
| pK18-4700 | Homologous recombination vector for C_04700 gene | Tsingke |
| pET28a | Prokaryotic expression | Lab stock |
| pDSK-GFPuv | Psa gene expression vector | Lab stock |
| **Primers** | **Sequences（5'-3'）** |  |
| P0F | CTGCAACAGGCGACGGCGAGGC | This study |
| P6R | CATAGGCTTCTGGTTTTCTTCCTGATCC |  |
| CL2-F | CGTTACCCGTTTCTTGGAT | This study |
| CL2-R | ACCGATGATAGCGTGCC |  |
| CL45-F | ATGGCTGTGCCTGGAAA | This study |
| CL45-R | CCACGAGCAGTCGGATTT |  |
| CLI56-F | TCGTGGAGGCGGTACTAAG | This study |
| CLI56-R | AGCAGAAACCCGAACAC |  |
| CL6-F | GTTAGTTGCGTTTGATTGCG | This study |
| CL6-R | TCAGGCTCTTGAGATGATTACTTT |  |
| CL7-F | AGTCTATCGGCGGGGT | This study |
| CL7-R | GCCTGTGCTCATCGTTC |  |
| hrpL-F | AGCCGGGTTATGTTCGC | This study |
| hrpL-R | TTGAGTCGAGGATCACAATCT |  |
| NLuc-NO | TATGACATGATTACGAATTCAGCATCCGCCCAATGCTC | This study |
| NLuc- NI | ACCACCAGAGCCACCGCCACCAGAGCCACCGTAACTGATACCTTTAGCGTTCGTC |  |
| NLuc-CO | GCCTGCAGGTCGACTCTAGACGTCTGGTTATTGGTGCCTTGC | This study |
| NLuc- CI | TTCTGGCGTAAATTATTTCTGATTGCCCCCTC |  |
| hrpANLuc-F | GGTGGCTCTGGTGGCGGTGGCTCTGGTGGTATGGTCTTCACACTCGAAGATTTC | This study |
| hrpANLuc-R | TCAGAAATAATTTACGCCAGAATGCGTTCG |  |
| SacB-F | GCAAACACTGGAACTGAAGATGG | This study |
| SacB-R | TTCCTTTCGCTTGAGGTACAGC |  |
| PK18-F | GAGTCAGTGAGCGAGGAAGCGGAAGAG | This study |
| PK18-R | TGTGCTGCAAGGCGATTAAGTTGGGTAA |  |
| Tn seq-F | CCCTTCCCGCTTCAGTGAC | This study |
| Tn seq-R | GTGCTTTACGGTATCGCCG |  |
| 2,SAM | GCGTAGAGGATCTGAAGATCAGC | This study |
| lac-P | TTATGCTTCCGGCTCGTATG |  |
| 22735NO | TATGACATGATTACGAATTCACGTCAGGCCATTTCGCAGA | This study |
| 22735NI | CTTCTTAGAGCCACCGCCACCAGAGCCACCGCGCTGAGCCTCCAGAGATGA |  |
| 22735CO | GCCTGCAGGTCGACTCTAGACCGTCCTGGCGAAAAGCTCTA | This study |
| 22735CI | GGTGGCTCTGGTGGCGGTGGCTCTAAGAAGTGCGTCGAAATACCGAAACGTTCT |  |
| M13F | GTTTTCCCAGTCACGAC | This study |
| M13R | CAGGAAACAGCTATGAC |  |
| GBD-F | GTGGCCCAATACGCAAACCG | This study |
| GBD-R | CTGCAAGGCGATTAAGTTGGGT |  |
| 4700RT-F | GCAGAACCTGACCCGCTACG | This study |
| 4700RT-R | GTGCGCCAATGCCCATG |  |
| 22735RT-F | TCATAAGGCCCAAACCCACC | This study |
| 22735RT-R | GCGTGACTCAATGCCTGTTC |  |
| 22735ORF-F | GGAATTCCATATGCGTTACGCGCTCGAT | This study |
| 22735ORF-R | CGGGATCCTCACAGATCCTCTTCAGAGATGAGTTTCTGCTCGCTAGTGCTCTTTTTTATCTC |  |
